# Calcium Peroxide Addition Suppresses ARG Dissemination by Modulating Host Succession and MGE-Mediated Horizontal Transfer During Swine Manure Composting

**DOI:** 10.3390/microorganisms14102294

**Published:** 2026-10-09

**Authors:** Wenxi Yang, Yuqiong Wang, Ting Lan, Tianxiang Wang, Lilong Luo, Liming Chen, Yunhang Gao, Changlong Gou

**Affiliations:** 1 College of Animal Science and Technology, Inner Mongolia Minzu University, Tongliao 028000, China; yangwenxi0926@163.com (W.Y.); wangyuq2015@163.com (Y.W.); lanting0430@163.com (T.L.); 15648504855@163.com (T.W.); luolilong0521@163.com (L.L.); chenliming1215@163.com (L.C.); 2 College of Animal Science and Technology, Jilin Agricultural University, Changchun 130118, China

**Keywords:** composting, swine manure, antibiotic resistance genes, mobile genetic elements, calcium peroxide

## Abstract

Applying exogenous additives during composting can help to effectively mitigate the environmental dissemination of antibiotic resistance genes (ARGs). Calcium peroxide (CaO_2_)—a strong oxidizing agent—has achieved significant efficacy in degrading various pollutants. This study aims to evaluate the effects of CaO_2_ at varying doses (0, 1% and 3% DW) on the variation in ARGs, mobile genetic elements (MGEs), and bacterial community succession during swine manure composting. Compared to the control (CK), adding 1% and 3% CaO_2_ reduced total ARGs and MGEs by 50.29% and 64.62% in the LCP and by 68.96% and 79.74% in the HCP treatment, respectively. Adding 1% and 3% CaO_2_ significantly increased the relative abundances (RAs) of *Actinobacteriota* and *Firmicutes* while decreasing those of *Proteobacteria* and *Bacteroidetes* during the thermophilic phase, especially under the high-dose treatment. Redundancy analysis (RDA) revealed that MGEs accounted for 54.20% of the total variation in ARGs, followed by environmental factors (30.3%). Network analysis further revealed that 1% and 3% CaO_2_ treatments disrupted the association between ARGs and their host bacteria. Overall, adding CaO_2_ can reduce the risks of spreading ARGs in compost products.

## 1. Introduction

Antibiotics are frequently administered in intensive livestock operations to improve growth performance and control infectious diseases. It has been documented that the total antibiotic consumption in global animal husbandry amounts to 99,502 tons and is forecast to rise to 107,472 tons by 2030 [1]. The majority of antibiotics are rarely absorbed by the animal’s intestinal tract and are excreted in feces as the parent compound or as metabolites [2]. These unmetabolized antibiotics can promote the proliferation of antibiotic-resistant bacteria (ARB) and accelerate the dissemination of ARGs, thus making livestock manure an important reservoir of antibiotics, ARB, and ARGs [3,4]. When livestock manure is applied as fertilizer, it facilitates the transfer and spread of ARB and ARGs into the environment via horizontal gene transfer (HGT) [5,6], which ultimately accumulates in animals and humans through the food chain, thereby posing substantial threats to both animal and human health. In particular, when ARGs are transferred to human pathogens via MGEs [7], they can trigger drug-resistant infections and render conventional antibiotic therapies ineffective. Globally, 700,000 people die each year owing to antibiotic resistance [8]. Therefore, mitigating ARG contamination in livestock manure is an urgent priority.

Composting is widely acknowledged as one of the most cost-effective and efficient strategies for the safe disposal and bioremediation of livestock manure [9]. Prior studies have shown that the composting process is capable of reducing ARGs, degrading various antibiotics, and eliminating pathogenic bacteria [10]. Furthermore, several studies have demonstrated that exogenous additives such as biochar [11], zeolite [12], and microbial agents [13] can enhance the elimination of antibiotics and ARGs and improve compost quality. However, the use of some exogenous additives in composting still presents challenges, including high cost, potential secondary pollution, and operational complexity, among others. Therefore, it is crucial to develop eco-friendly, cost-effective, and practical exogenous additives for further alleviating the spread of ARGs during composting.

Calcium peroxide (CaO_2_)—also known as solid hydrogen peroxide (H_2_O_2_)—gradually releases H_2_O_2_ and O_2_ under humid conditions (Equations (1) and (2)) without undergoing a disproportionation reaction [14]. It is widely applied as a safe and economical oxidizing agent for soil remediation and water improvement [14,15,16,17]. Studies show that CaO_2_ can promote microbial metabolism [18], enhance antibiotic degradation [19], and facilitate heavy metal passivation [20]. In recent years, CaO_2_ has been increasingly introduced into composting systems to enhance the harmless treatment and resource recovery of organic wastes. Bao et al. [21] added CaO_2_ to a sewage sludge composting system, revealing that 10% CaO_2_ addition enhanced organic matter degradation and increased humic substances by 24.20%. Lu et al. [22] introduced CaO_2_ into a straw–sludge composting system, revealing that 1% CaO_2_ addition increased reactive oxygen species (ROS) content and reduced the RAs of ARGs by 19.02%. Bao et al. [23] reported that adding 10% CaO_2_ to the sewage sludge composting process could effectively passivate heavy metals (Cu, Ni, and Zn) and significantly reduce the RAs of total ARGs by 79.40%. Other studies show that CaO_2_ addition enhances temperature increase, stimulates microbial metabolism, and promotes MGE reduction during composting [22,24,25]. However, limited research exists regarding the effect of CaO_2_ on ARGs during swine manure composting. Therefore, CaO_2_ holds considerable potential as an effective strategy for mitigating ARGs in the composting process.

CaO_2_ + H_2_O → O_2_ + Ca (OH)_2_ (1)

CaO_2_ + H_2_O → H_2_O_2_ + Ca (OH)_2_ (2)

Based on the above considerations, we formulated three hypotheses for this study: (i) CaO_2_ addition reduces the RAs of ARGs and MGEs in a dose-dependent manner during swine manure composting; (ii) CaO_2_ addition is associated with a reduced potential for MGE-mediated HGT; (iii) CaO_2_ addition reshapes the bacterial community and reduces potential ARG host populations, mitigating ARG dissemination risks in compost.

To test these hypotheses, the present study was conducted to investigate the influence of CaO_2_ on ARGs and MGEs during swine manure composting. High-throughput quantitative PCR (HT-qPCR) was used to quantify the variations in ARGs and MGEs, while 16S rRNA high-throughput sequencing was employed to characterize bacterial community succession and verify the influence of CaO_2_ on the RAs of ARGs and MGEs. The findings can provide a novel strategy for mitigating ARGs during composting and promote the safe and sustainable treatment of livestock and poultry manure.

## 2. Materials and Methods

### 2.1. Composting Setup and Sampling

Corn stalks and swine manure, serving as raw materials for composting, were obtained from a cornfield and a pig farm in Tongliao, Inner Mongolia, China, respectively. Industrial-grade CaO_2_ (75% purity) was purchased from Ningbo Actmix Rubber Chemicals Co., Ltd. (Ningbo, China). Physicochemical characteristics of the raw materials are listed in Table 1. Three treatments were established by supplementing the composting material with different proportions of CaO_2_: 0%, 1%, and 3% (CK, LCP, and HCP treatments, respectively) on a dry-weight basis, with 3 replicates per treatment. The swine manure and corn stalks were uniformly blended at 4:1 (*w*/*w*) to a C/N ratio of 30:1, with the moisture content adjusted to 60%. The mixed raw materials were composted continuously for 30 days in a self-constructed composting reactor (60 L working volume) with two holes (2 cm × 2 cm) on each side to facilitate natural ventilation, as described in a previous study [26]. Compost samples were collected at the initial (0 d), thermophilic (3 d), cooling (10 d), and maturation stages (30 d). A 100 g portion of each compost sample was randomly collected from three locations in each reactor (5 cm away from the top, the middle layer, and 5 cm away from the bottom) and homogenized by the quartering method to obtain a representative sample. One portion of the sample was stored at 4 °C, and the other portion was stored at −80 °C for subsequent analyses.

### 2.2. Physicochemical Analysis

The composting and ambient temperatures were monitored using a digital thermometer twice per day. Moisture content was measured by oven-drying at 105 °C for 24 h. The pH was measured in a 1:10 (*w*/*v*) water extract using a pH electrode. TOC and TN were determined by the K_2_Cr_2_O_7_ oxidation method [27] and the Kjeldahl method [28], respectively.

### 2.3. DNA Extraction and Target Gene Detection

Total DNA was extracted with the OMEGA Soil DNA Kit (Omega Bio-Tek, Norcross, GA, USA), and its concentration was determined using a NanoDrop NC-2000 spectrophotometer. High-quality DNA was used for subsequent analysis of HT-qPCR and bacterial community.

A total of 36 ARGs (including 9 tetracycline subtypes, 3 sulfonamide subtypes, 5 aminoglycoside subtypes, 5 MLSB subtypes, 2 trimethoprim subtypes, 2 vancomycin subtypes, 2 beta-lactam subtypes, 1 multidrug subtype, 3 fluoroquinolone subtypes, 4 other subtypes) and 5 MGEs (*intI1*, *intI2*, *IS26*, *IS613*, and *ISCR1*) were detected using HT-qPCR with the WaferGen SmartChip Real-Time PCR System. The RAs of ARGs and MGEs were normalized based on the copy number of the 16S rRNA gene. The specific primer sequences and reaction conditions for HT-qPCR are shown in S1 and Table S1.

### 2.4. 16S rRNA Gene Sequencing

Bacterial community sequencing was performed on the Illumina NovaSeq platform (Personal Bio. Co., Ltd., Shanghai, China). The V3–V4 hypervariable region of the bacterial 16S rRNA gene was amplified with the primer pair 338F-806R. Raw sequencing data were subjected to quality control and merging using QIIME2 [29], and the resulting non-singleton amplicon sequence variants (ASVs) were taxonomically annotated with the classify-sklearn naive Bayes classifier against the Greengenes database (release 13_8 99% OTUs reference sequences) [30].

### 2.5. Statistical Analyses

Statistical analysis of variance (ANOVA) was performed using PASW Statistics 18.0 (IBM, Armonk, NY, USA). Variations in ARGs and MGEs were visualized with GraphPad Prism 8. Heatmap analysis and principal coordinates analysis (PCoA) were visualized with R 4.3.1. RDA was carried out in Canoco 5. Forward selection was performed on 16 explanatory variables (5 MGEs, 6 environmental factors, 5 dominant bacterial phyla), with significance evaluated by Monte Carlo permutation tests (999 permutations). Variation explained by each variable block was calculated from conditional effects of retained terms. Partial least squares path modeling (PLS-PM) quantified direct and indirect effects of environmental factors, MGEs and bacteria on ARG dissemination. Model fit was evaluated by the goodness-of-fit (GoF) index, and path significance was assessed by bootstrap resampling (1000 iterations). Analysis was performed using the plspm package in R version 4.3.1. Co-occurrence networks were constructed for the top 30 bacterial genera, ARGs and MGEs. Spearman’s rank correlations were computed in R 4.3.1, and positive correlations (r > 0.6, *p* < 0.05) were retained to build the adjacency matrix, which was imported into Gephi 0.10.1 for visualization and topological analysis (average degree; weighted Louvain modularity, resolution = 1.0). Linear discriminant analysis effect size (LEfSe) was implemented with the one-against-all multi-class strategy, using the Mann–Whitney U test (*p* < 0.05) and an LDA effect size threshold of 2.0, and the visualization was generated using R 4.3.1.

## 3. Results and Discussion

### 3.1. Changes in ARGs

**Figure 1:**
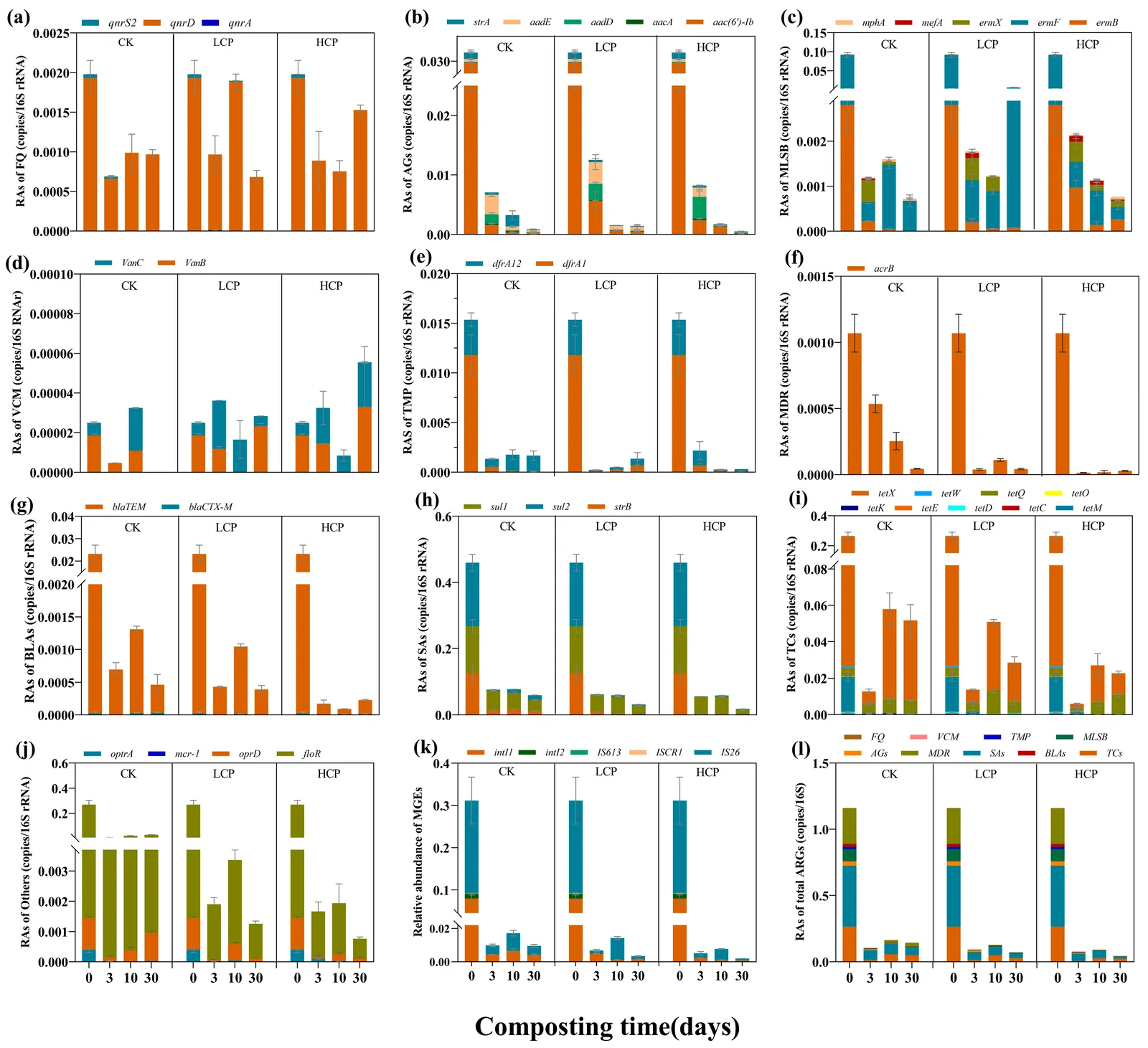
Changes in relative abundance of ARGs and MGEs during composting. (**a**) Fluoroquinolone resistance genes; (**b**) aminoglycoside resistance genes; (**c**) macrolide–lincosamide–streptogramin B resistance genes (MLSB); (**d**) vancomycin resistance genes; (**e**) trimethoprim resistance genes; (**f**) multidrug resistance genes; (**g**) beta-lactam resistance genes; (**h**) sulfonamide resistance genes; (**i**) tetracycline resistance genes; (**j**) other resistance genes; (**k**) total abundance of the 5 MGE types; (**l**) total abundance of the 36 ARG types. The error bars represent the standard deviation of three repeated samples; the same applies hereinafter.

In this study, 36 ARG subtypes belonging to nine antibiotic classes were detected, with RAs ranging from 10^−6^ to 10^0^ gene copies/16s rRNA gene copy in the raw materials. Sulfonamide resistance genes were the most abundant ARGs (4.59 × 10^−1^ gene copies/16S rRNA copy; Figure 1 and Table S2), probably attributed to the extensive application and high excretion rates of sulfonamides in swine production [31,32]. After composting started, the total ARG RAs declined sharply to 0.10 in CK, 0.09 in LCP, and 0.08 in HCP during the thermophilic period (day 3). Similar findings have been reported, indicating that elevated temperatures can efficiently impair host bacteria and deactivate ARGs [33]. Subsequently, the total ARG RAs declined further until the composting was completed. At the completion of composting, the reductions in total ARG RAs in the LCP and HCP treatments were significantly greater than those in CK, indicating that composting can effectively eliminate ARGs, while CaO_2_ addition can further enhance their removal, with the most prominent effect detected in the HCP treatment group. Previous studies show that adding CaO_2_ into sludge composting systems facilitates ARG degradation via generating hydroxyl radicals (•OH) and ROS [23], along with decreasing the abundance of host bacteria [22]. The ROS-related pathway is presented as a literature-based early-stage hypothetical effect. Day-30 ARG shifts are attributed to persistent physicochemical alterations and microbial succession triggered by initial amendment, rather than residual short-lived ROS.

Regarding ARG categories, at the completion of composting, the RAs in different ARG categories generally decreased compared with the initial materials, except for vancomycin resistance genes, which increased (Figure 1d). Specifically, the RAs of trimethoprim, beta-lactam, sulfonamide, multidrug, and tetracycline resistance genes gradually decreased with increasing CaO_2_ dosage. These findings indicate that CaO_2_ addition enhances the removal of ARGs in most categories during composting. However, after composting, the RAs of MLSB and fluoroquinolone resistance genes (FQRGs) in the HCP treatment were higher than those in CK (Figure 1a,c). The RAs of MLSB resistance genes and FQRGs were 1.06- and 1.58-fold higher, respectively. Furthermore, FQ resistance genes exhibited a 1.58-fold increase under high-dose treatment, suggesting that certain ARGs may persist or even accumulate despite treatment.

Figure S1 illustrates the changes in the RA of each ARG after composting. Compared with the raw materials, most ARGs exhibited decreased RAs to varying extents (CK: 2.1–100%, LCP: 12.42–100%, and HCP: 20.99–100%), except for several ARGs that increased, including *tetQ*, *tetK*, and *vanB* in LCP and HCP; *blaCTX-M* in CK; and *aadD* and *vanC* in HCP. After composting, compared to the CK treatment, 12 ARGs (*tetE*, *tetM*, *tetW*, *tetX*, *strB*, *sul1*, *sul2*, *floR*, *dfrA12*, *blaTEM*, *optrA* and *qnrD*) showed significant decreases in the LCP treatment. Similarly, 17 ARGs (*tetC*, *tetD*, *tetE*, *tetM*, *tetX*, *tetW*, *strB*, *sul1*, *sul2*, *floR*, *ermF*, *aadE*, *dfrA12*, *blaTEM*, *blaCTX-M*, *aac*(*6*′)*-Ib* and *optrA*) in the HCP treatment exhibited even more significant decreases. These findings suggest that high-dose CaO_2_ treatment substantially reduces the RAs of a larger number of ARG subtypes. Notably, the increased abundance of *vanC* likely accompanied the enrichment of its host *Enterococcus* spp., since *vanC* is a chromosomally borne intrinsic resistance determinant.

### 3.2. Changes in MGEs

MGEs are critical mediators for the HGT of ARGs in the environment [34]. In the present study, five MGEs (*intI1*, *intI2*, *ISCR1*, *IS26*, and *IS613*) were detected to assess the dissemination ability of 36 ARGs (Figure 1k). The total RAs of MGEs decreased sharply to bottom values during the thermophilic period (0.01 in CK, 0.007 in LCP, and 0.005 in HCP) and then declined further until the end of composting. These findings are consistent with Wan et al. [7], who reported that high composting temperatures contribute significantly to MGE reduction. At the end of composting, the total RAs of MGEs declined to 0.01, 0.003, and 0.002 in CK, LCP, and HCP treatments, respectively. Compared with CK, the greater reduction in MGEs in LCP and HCP treatments suggests that CaO_2_ addition may remove more ARGs and hinder their horizontal transfer via MGEs.

Among MGEs, *intI1* and *intI2* are detected most frequently in the environment and can capture environmental ARGs, recombining them into integron gene cassettes [35,36]. Additionally, IS family elements are often found adjacent to ARGs or contribute to ARG mobilization [37,38]. Among the detected MGEs, *IS26* was the most dominant MGE, with 2.19 × 10^−1^ gene copies/16S rRNA copy. Figure S2 shows the changes in the RA of each MGE at the completion of composting. Compared with the raw materials, all MGEs exhibited decreased RAs to varying degrees of 94.71–99.32%, 96.32–100%, and 95.81–99.79% in CK, LCP, and HCP treatments, respectively. After composting, compared to the CK treatment, the RAs of all four MGEs decreased to varying degrees, except for *IS613*, which exhibited a slight, non-significant increase in HCP. These findings indicate that CaO_2_ addition can effectively reduce most MGEs.

### 3.3. Changes in Bacterial Community

**Figure 2:**
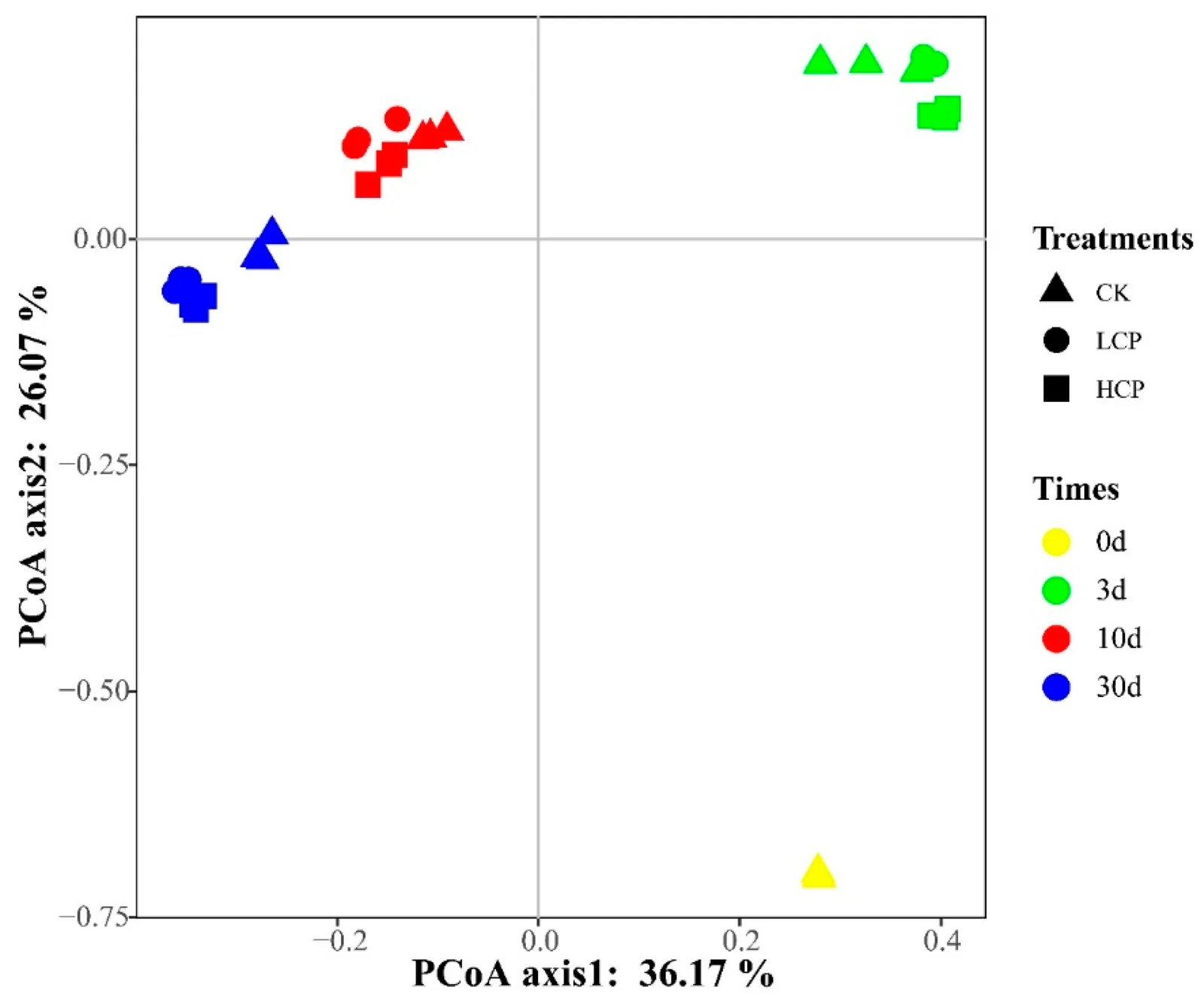
Principal coordinate analysis of bacterial communities during swine manure composting.

PCoA was employed to evaluate the differences in bacterial community composition across various samples. Axis 1 and axis 2 accounted for 62.24% of the variance (Figure 2). The bacterial communities were divided into four clusters by composting periods, demonstrating that composting period plays a greater role than CaO_2_ addition in shaping the microbial community. We further conducted PERMANOVA on the same Bray–Curtis dissimilarity matrix (ASV-level table, 9999 permutations). For the two-way model (composting stage: days 3, 10, 30; CaO_2_ dose: CK, LCP, HCP; *n* = 27), composting stage explained 58.4% of community variation (F = 15.47, *p* < 0.001) and CaO_2_ dose explained 20.3% (F = 2.80, *p* = 0.008; residual = 21.3%), supporting the PCoA-derived observation.

**Figure 3:**
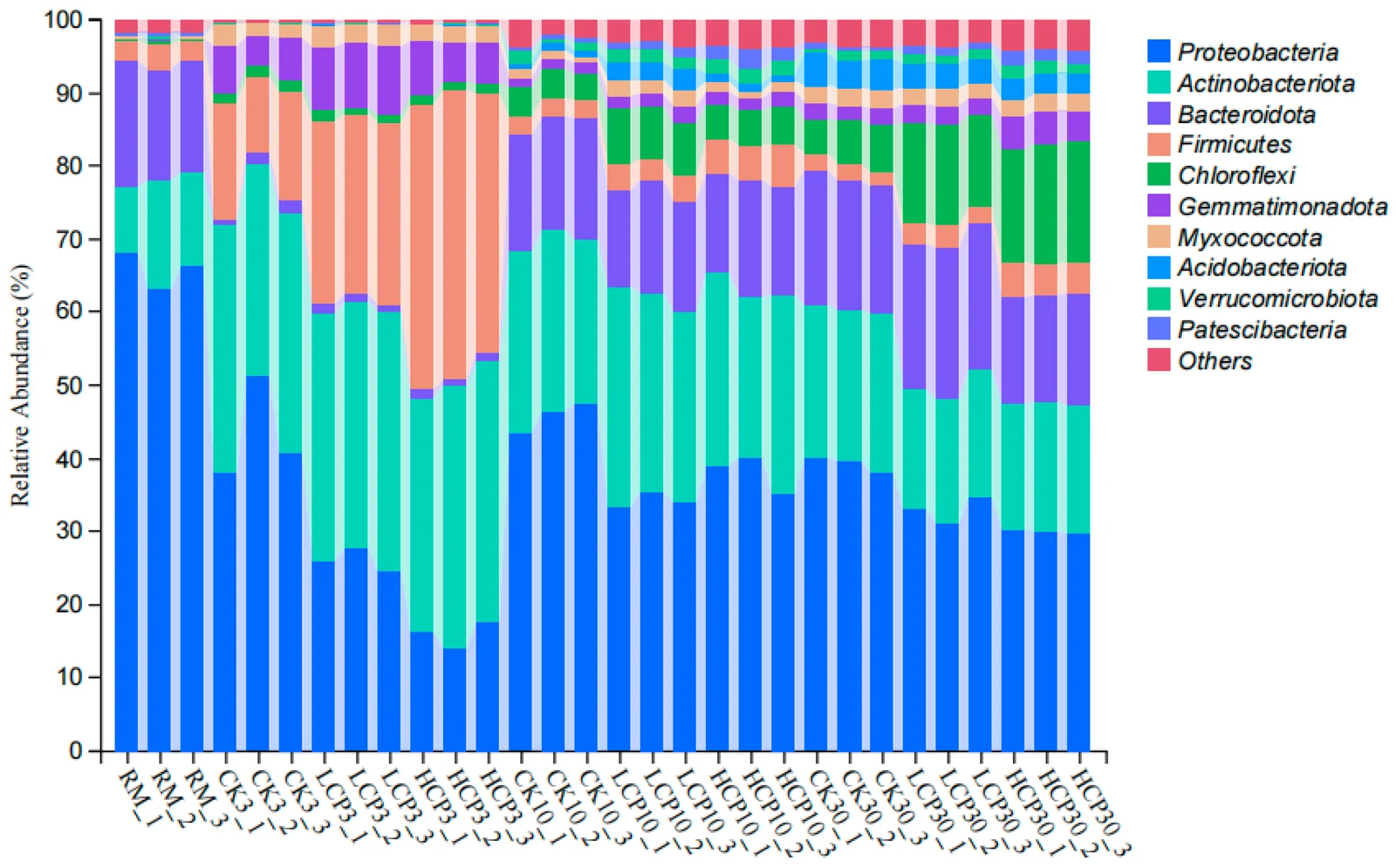
Evolution of the bacterial community (phylum level) in swine manure during composting.

Dynamic changes in the RAs of bacteria at the phylum and genus levels during composting are presented in Figure 3 and Figure S3. *Proteobacteria* (14.01–68.16%), *Firmicutes* (2.20–39.67%), *Actinobacteriota* (9.08–35.97%), and *Bacteroidota* (0.64–20.49%) were the four most dominant bacterial phyla during composting. In raw materials, *Proteobacteria* and *Bacteroidota* were the most abundant bacterial phyla (65.93% and 15.91%, respectively), but their RAs decreased rapidly during the thermophilic period to 43.46% and 1.26%, 26.11% and 1.12%, 15.96% and 0.93% in CK, LCP, and HCP, respectively. This decline was also paralleled by an order-of-magnitude reduction in dominant genera, including *Acinetobacter*, *Stenotrophomonas*, and *Flavobacterium*, which are potential hosts of ARGs and pathogens [26,39,40]. Subsequently, *Proteobacteria* rebounded to 39.32%, 33.08% and 30.06%, and *Bacteroidota* to 14.90%, 20.26% and 16.57% for the CK, LCP and HCP groups, respectively, by the end of composting. Compared to CK, *Proteobacteria* decreased by 16.69% and 23.53% in LCP and HCP, respectively, while *Bacteroidota* decreased by 16.57% in HCP. This reduction is likely attributed to lower RAs in *Proteobacteria* genera *Steroidobacter* and *Pseudoxanthomonas*, and *Bacteroidota* genera *Puia* and *Niabella*, most of which are host bacteria for ARGs [41].

**Figure 4:**
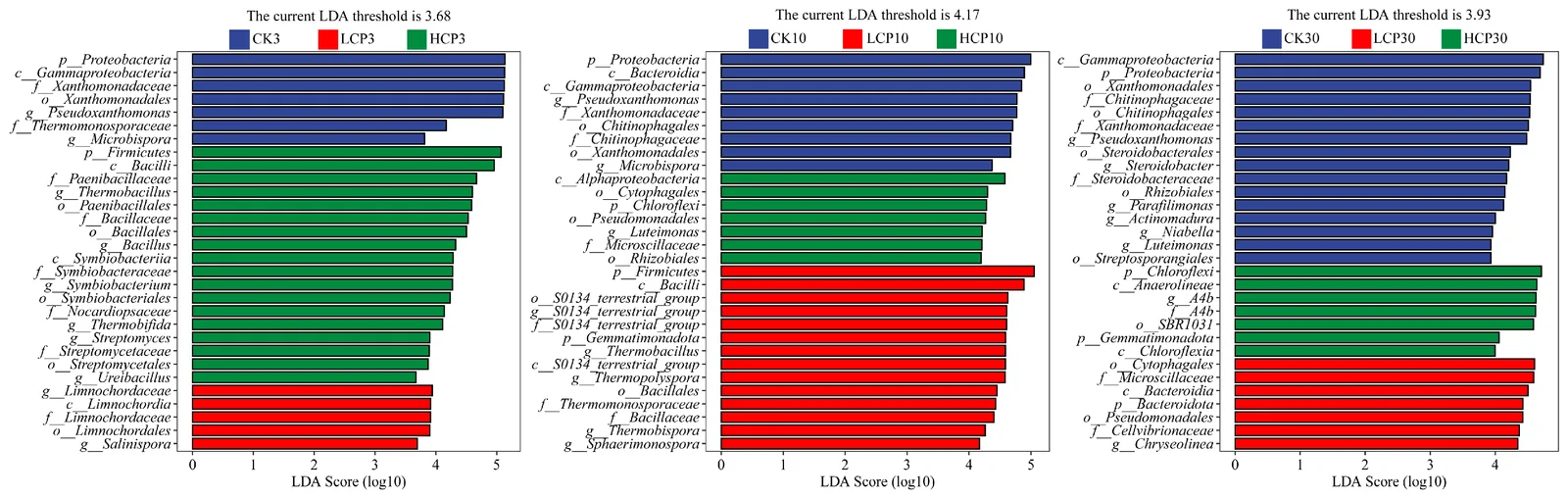
Linear discriminant analysis effect size (LEfSe) analysis showing the LDA score of discriminative bacterial taxa across different treatments during composting.

To further identify statistically significant indicator taxa responding to CaO_2_ addition at each composting stage, LEfSe analysis was performed (LDA > 2, *p* < 0.05, Figure 4). Consistent with the above relative abundance observations, LEfSe revealed distinct biomarker taxa enriched in CK, LCP and HCP at day 3, day 10 and day 30. At the thermophilic stage (day 3), *Proteobacteria*-related taxa were the biomarkers of CK, whereas *Firmicutes* including *Thermobacillus*, *Bacillus* and *Symbiobacterium* were significantly enriched as indicator taxa in HCP. On day 10, *Firmicutes* became the signature taxa for LCP, while *Alphaproteobacteria* and *Chloroflexi* were biomarkers in HCP. At the maturation stage (day 30), *Gammaproteobacteria*-related genera (*Pseudoxanthomonas*, *Steroidobacter*) remained characteristic taxa in CK; *Chloroflexi* were enriched in HCP, and *Bacteroidota* taxa were biomarkers for LCP. These biomarker taxa verified that CaO_2_ addition reshaped bacterial community succession and selectively suppressed ARG-host taxa affiliated with *Proteobacteria.* Therefore, this indicates that higher CaO_2_ additions effectively reduce the potential host and pathogenic bacteria during composting, thereby decreasing ARG prevalence.

Conversely, *Firmicutes* and *Actinobacteriota* increased rapidly, reaching peak values of 13.67% and 31.92%, 24.77% and 34.39%, and 38.10% and 34.67% in CK, LCP, and HCP, respectively, on day 3, and then decreased to 2.29–4.32% and 16.88–20.99% after composting. Compared to CK, low and high CaO_2_ doses increased the RAs of *Firmicutes* by 1.81- and 2.79-fold, and *Actinobacteriota* by 7.74% and 8.61%, respectively, during the thermophilic period. These changes were also reflected by larger increases in the RAs of *Firmicutes* genus members, such as Thermobacillus, Bacillus, Symbiobacterium, and Limnochordaceae (Figure 4), and modest increases in the dominant *Actinobacteriota* genera (*Sphaerimonospora*, *Streptomyces*, and *Corynebacterium*). Previous studies demonstrate that *Firmicutes* and *Actinobacteriota* are the dominant bacterial phyla during the thermophilic period of composting, because most of their genus members possess the ability to withstand high temperatures and degrade lignocellulose [42,43]. These findings indicate that CaO_2_ additions accelerate the degradation of recalcitrant lignocellulose in the compost pile, promoting composting maturation.

Specifically, the *Chloroflexi* gradually increased from 0.44% at day 0 to 5.70%, 13.33%, and 16.27% in CK, LCP, and HCP, respectively, by day 30. A similar result was also found by Peng et al. [44], who reported *Chloroflexi* enrichment during the cooling and maturation phases of kitchen waste composting. Compared to CK, the RAs of *Chloroflexi* in LCP and HCP increased by 2.34- and 2.85-fold, respectively, at the end of composting. This pattern was also demonstrated by significantly higher RAs of its dominant genera (*JG30-KF-CM45*, *A4b*, and *FFCH7168*) in LCP and HCP treatments. Genera *A4b* and *JG30-KF-CM45* participate actively in nitrogen transformation during composting [45]. These findings indicate that CaO_2_ addition may enhance the quality of compost products by improving ammonia nitrogen conversion and nitrate nitrogen formation (Figure S4).

### 3.4. Relationships Among ARGs, MGEs, Bacterial Community, and Environmental Factors

**Figure 5:**
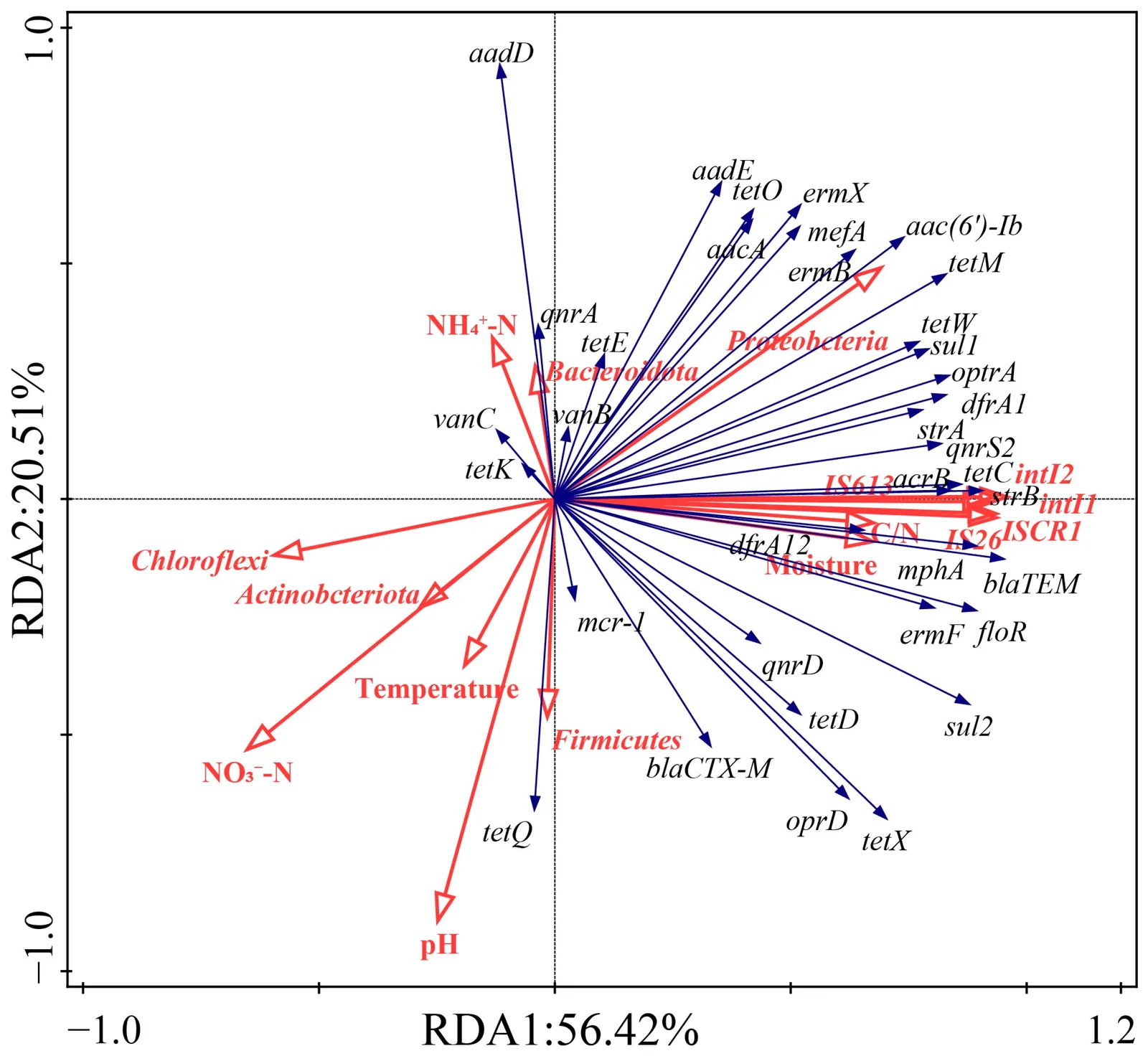
Redundancy analysis of the relationships between bacterial communities, MGEs, environmental factors, and ARGs during swine manure composting.

**Figure 6:**
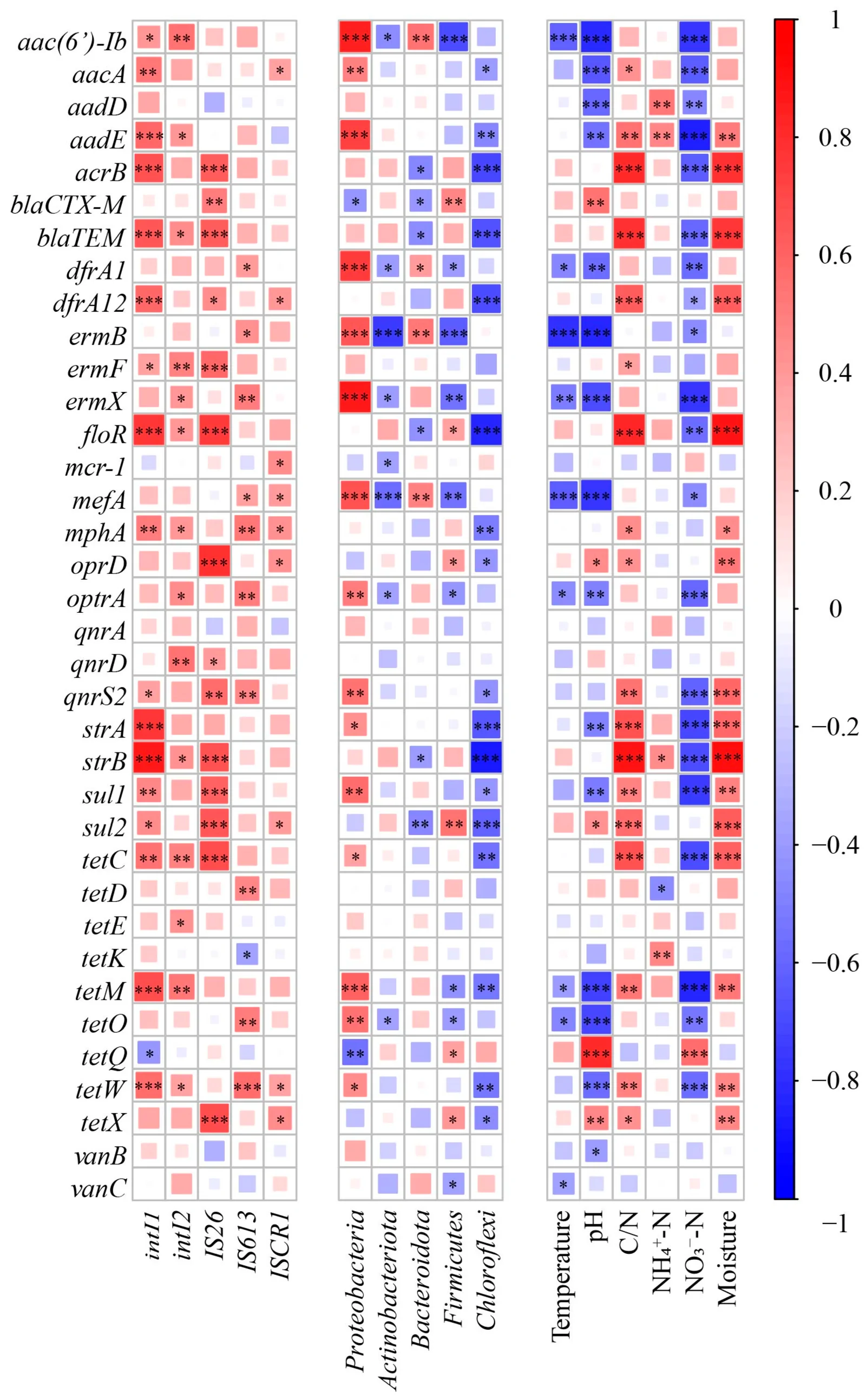
Spearman correlation analysis of ARGs, MGEs, the bacterial community, and environmental factors. Symbols “*”, “**”, and “***” indicate significance at the *p* < 0.05, *p* < 0.01, and *p* < 0.001 levels, respectively.

RDA was conducted to investigate the contributions of MGEs (*intI1*, *intI2*, *IS613*, *IS26*, and *ISCR1*), the bacterial community (top five phyla) (Figure 5 and Figure 6) and environmental factors (temperature, pH, C/N, moisture, NH_4_^+^-N, and NO_3_^−^-N, Figure S4) to the changes in ARGs. RDA1 and RDA2 explained 76.93% of the total variation in ARGs. MGEs were the primary contributors to ARG changes (54.2%), followed by environmental factors (30.3%) and the bacterial community (11.1%). Variations in the ARGs were mainly affected by the HGT mediated via MGEs [46]. Except for *aadD*, *qnrA*, *tetK*, *tetQ*, *vanB*, and *vanC*, all ARG subtypes showed significant positive correlations with one or more MGEs. Among the MGEs, intI1 (52.1%) accounted for the most ARG variations and exhibited significant positive correlations (*p* < 0.05) with 17 ARG subtypes (*aac*(*6*′)*-Ib*, *aacA*, *aadE*, *acrB*, *blaCTX-M*, *dfrA12*, *ermF*, *floR*, *mphA*, *qnrS2*, *strA*, *strB*, *sul1*, *sul2*, *tetC*, *tetM*, and *tetW*). This association most likely reflects the functional role of intI1 in HGT rather than general contamination, since intI1 is among the most frequently detected MGEs in environmental samples and can capture and recombine environmental ARGs into integron gene cassettes [35,36]; it is a statistical association and not proof of HGT. As previously mentioned, the abundance of intI1 in HCP decreased markedly at the end of composting. Therefore, decreases in the RAs of these ARGs may be attributed to the decreased RAs of *intI1* after composting.

Environmental factors were another prominent variable accounting for the variations in ARGs. Among the environmental factors, pH explained 18.5% (F = 17.0, *p* = 0.002) of ARG variance. Numerous previous studies confirm that alkaline pH inhibits HGT and induces host bacterial apoptosis, thereby affecting ARG migration during composting [47,48]. In this study, 15 ARG subtypes exhibited significant negative correlations with pH. Specifically, ARG subtypes, including *aac*(*6*′)*-Ib*, *aadE*, *optrA*, *strA*, *sul1*, and *tetM*, showed substantially lower RAs in the HCP treatment with higher pH, which further supports this observation.

**Figure 7:**
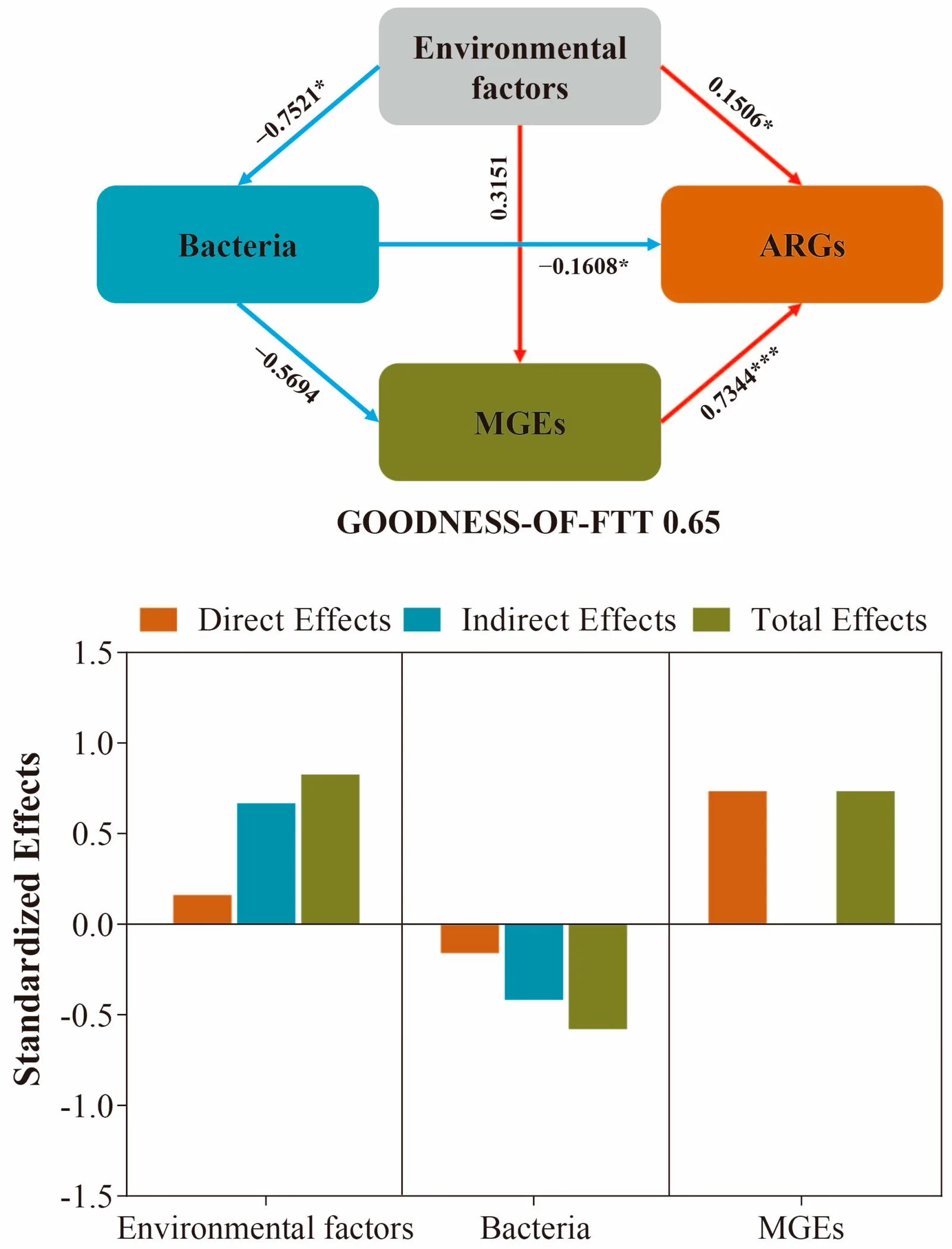
PLS-PM showing the direct and indirect effects of different driving factors on ARGs. Numbers adjacent to arrows indicate standardized path coefficients. Symbols “*” and “***” indicate significance at the *p* < 0.05 and *p* < 0.001 levels, respectively.

We further analyzed the direct and indirect effects of different driving factors on ARGs using a structural equation model constructed by PLS-PM (Figure 7). The results revealed that both MGEs and environmental factors had significant direct effects on ARGs, whereas the bacterial community influenced ARGs indirectly. This indirect effect was mediated mainly through MGEs (indirect effect = −0.418 vs. a negligible direct effect of −0.1608), because the bacterial community supplies the cellular hosts carrying MGEs and their linked ARGs, whereas pH acts as a component of the environmental factors variable with a direct path to ARGs. MGEs exhibited a stronger direct effect on ARGs than environmental factors, and environmental factors also directly affected ARGs by suppressing the enrichment of MGEs. These findings indicate that CaO_2_ addition effectively reduces ARGs by lowering MGE levels and inhibiting the dissemination of ARGs.

**Figure 8:**
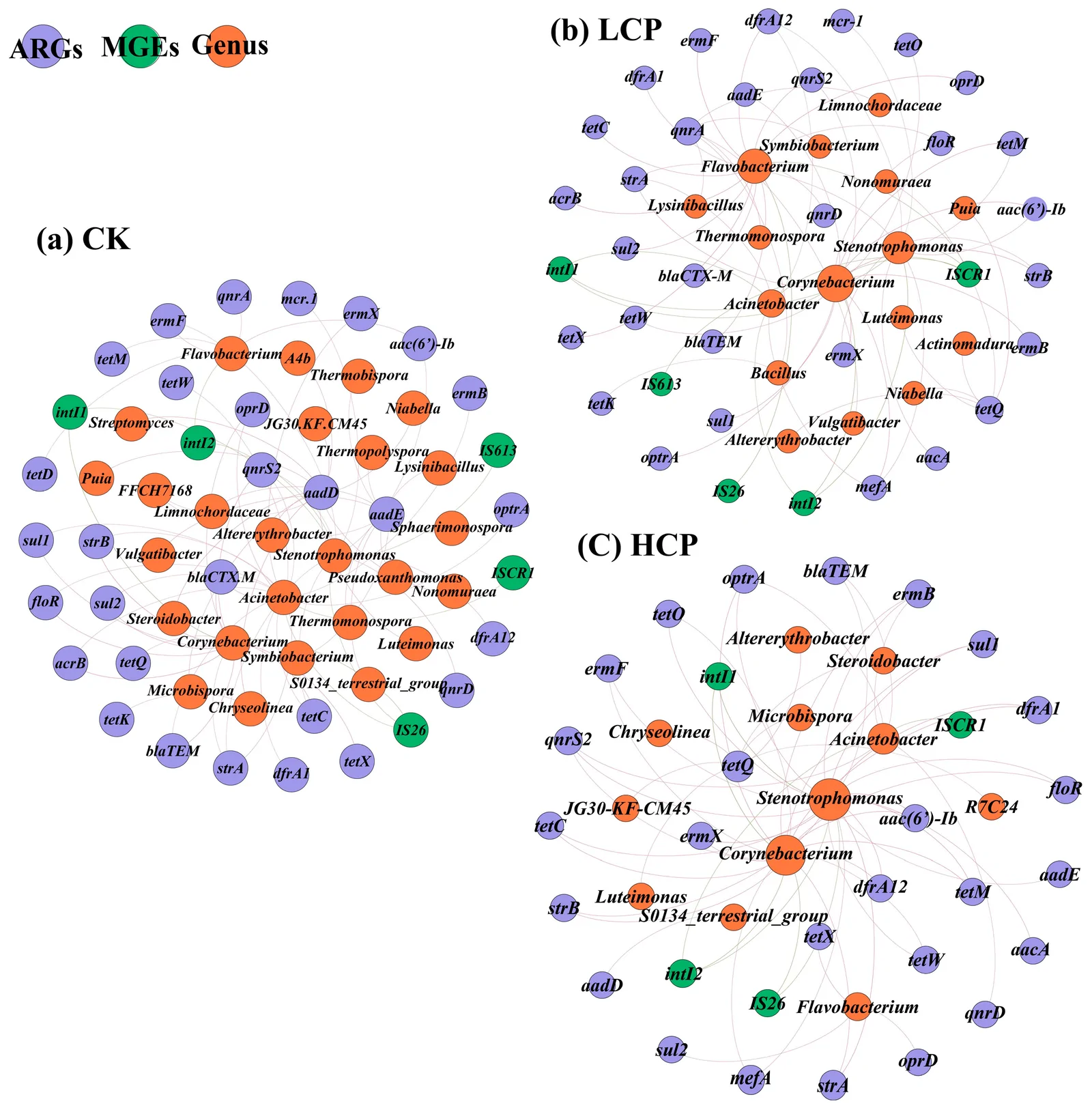
Network analysis showing the co-occurrence relationships among ARGs and MGEs and potential host bacteria (*p* < 0.05).

During swine manure composting with different CaO_2_ doses, network analysis was employed to investigate the co-occurrence profiles among ARGs, MGEs, and bacterial genera. Network analysis revealed 60 nodes (29 ARGs, 5 MGEs, and 26 genera) with 75 edges in CK, 51 nodes (30 ARGs, 5 MGEs, and 16 genera) with 64 edges in LCP, and 42 nodes (26 ARGs, 4 MGEs, and 12 genera) with 61 edges in HCP, respectively (Figure 8). Both node and edge numbers decreased with increasing CaO_2_ dose, indicating fewer significant co-occurrence relationships between ARGs/MGEs and bacterial genera. Among the retained nodes, however, the average degree increased from 2.50 (CK) and 2.51 (LCP) to 2.90 (HCP), while modularity declined from 0.606 (CK) to 0.541 (LCP) and 0.482 (HCP); the networks were thus simplified overall, but the remaining nodes became more densely interconnected and the original modular structure was progressively disrupted. The normalized topological metrics showed that HCP had the highest average degree (3.21), followed by LCP (2.78) and CK (2.73). Meanwhile, modularity decreased from 0.606 (CK) to 0.541 (LCP) and 0.482 (HCP). These results indicate stronger inter-connections among the retained nodes under high-dose treatment, accompanied by gradual disruption of the original modular structure of the co-occurrence network. *Corynebacterium*, *Stenotrophomonas*, and *Acinetobacter* were among the shared potential host genera of ARGs and MGEs across all treatments. Previous studies show that these three pathogenic bacteria are enriched in chicken and pig manure [39,49]. In the present study, at the end of composting, these three bacterial genera decreased to a relatively low level (<0.06%). In CK, *aadD* exhibited the highest number (12) of potential bacterial hosts, no co-occurring hosts were found in LCP, and only one remained in HCP. *Acinetobacter*, the most abundant potential host in CK, co-occurred with 16 ARGs and 2 MGEs. However, it was reduced to 3 ARGs with 2 MGEs in LCP and 5 ARGs with 2 MGEs in HCP. These findings indicate that CaO_2_ addition may disrupt the association between ARGs and their host bacteria.

## 4. Conclusions

In this study, three hypotheses concerning the effect of CaO_2_ addition on ARGs during swine manure composting were tested. Hypothesis (i) was largely supported: low- and high-dose CaO_2_ addition reduced total ARGs by 50.29% and 68.96%, and total MGEs by 64.62% and 79.74%, indicating a dose-dependent reduction, with only a few ARG subtypes deviating from this pattern. Hypothesis (ii) was supported: total MGE RAs in LCP and HCP decreased by 96.32–100% and 95.81–99.79%, and the ARG-MGE co-occurrence network contracted under high-dose CaO_2_ amendment. Hypothesis (iii) was supported: low- and high-dose CaO_2_ addition significantly increased the RAs of *Actinobacteriota* and *Firmicutes* while decreasing those of *Proteobacteria* and *Bacteroidetes* during the thermophilic phase, especially under the high-dose treatment. Overall, these findings offer a promising strategy for mitigating ARG-related hazards during swine manure composting.

## Figures and Tables

**Table 1:** Characteristics of the raw materials for composting.

Raw Materials	Moisture (%)	pH	TOC (g kg^−1^)	TN (g kg^−1^)
Swine manure	73.50 ± 0.21	7.14 ± 0.05	400.60 ± 4.68	20.10 ± 0.54
Corn stalk	8.49 ± 0.02	7.02 ± 0.02	421.11 ± 5.36	7.02 ± 0.03

## Data Availability

The original contributions presented in this study are included in the article/Supplementary Materials. Further inquiries can be directed to the corresponding author.

## Supplementary Materials

The following supporting information can be downloaded at https://www.mdpi.com/article/10.3390/microorganisms14102294/s1, Figure S1. RAs of each ARGs after composting; Figure S2: RAs of each MGEs after composting; Figure S3: Heatmap showing relative abundances of the top 30 genera in all samples; Figure S4: Changes in the physicochemical properties during composting; Table S1: Primer sequences for PCR; Table S2. The absolute quantification of ARGs, MGEs and 16S rRNA genes.
