# Supplementary material for: Calcium Peroxide Addition Suppresses ARG Dissemination by Modulating Host Succession and MGE-Mediated Horizontal Transfer During Swine Manure Composting
Source: Microorganisms. 2026 Oct 9;14(10):2294. doi: 10.3390/microorganisms14102294 (PMC13707557; doi:10.3390/microorganisms14102294)
Supplement: Supplementary file 1 [file microorganisms-14-02294-s001.zip › microorganisms-4544651-supplementary.pdf]

Calcium peroxide addition suppresses ARG dissemination by modulating host succession and MGE-mediated horizontal transfer during swine manure composting

Wenxi Yang [a], Yuqiong Wang [a], Ting Lan [a], Tianxiang Wang [a], Lilong Luo [a], Liming Chen [a], Yunhang Gao [b*], Changlong Gou [a*]

[a] College of Animal Science and Technology, Inner Mongolia Minzu University, Tongliao 028000, China;

[b] College of Animal Science and Technology, Jilin Agricultural University, Changchun, 130118, China

*Corresponding authors:

Correspondence: gcl1988@imun.edu.cn (C.G.); gaoyunhang@163.com (Y.G.)

S1. The primer reaction procedure

Total DNA was extracted using a FastDNA SPIN Kit for soil (MP Biomedicals, USA) following the manufacturer's instructions of microbial functional genes were performed Samples were loaded onto the SmartChip Multisample Nanodispenser (MSND) using a 64 (assays)×80(samples) array. The HT-qPCR array consisted of 36 primer sets. Each 100 nL reaction mixture contained 50 nL of 2× LightCycler 480 SYBR Green I Master Mix (Roche Inc., USA), 20 nL of a 2 ng/μl DNA template,1 nL of 0.1 mg/ml bovine serum albumin, 500 nM each of the forward and reverse primers, and 19 nL of nuclease-free PCR-grade water. For each primer set, amplification was conducted in triplicate and a non-template control was included. The thermal regimen included an initial denaturation at 95 °C for 10 min, followed by 40 cycles of denaturation at 95 °Cfor 30 s, annealing at 58 °C for 30 sand extension at 72 °C for 30 s, finally with melting curve analysis was autogenerated by the program [1].

Results of HT-qPCR were analyzed using the SmartChip qPCR software (version 2.7.0.1). Wells with multiple melting peaks or amplification efficiencies outside the range of 1.8-2.2 were not included in the analysis. A limit was set on the number of cycles necessary to observe a significant fluorescence signal (threshold cycle, Ct). Only samples exhibiting Ct < 31 cycles and having more than two replicates showing amplification were regarded as positive. Relative copy number was calculated according to the equation described in Ouyang et al. [2]. Relative abundance of microbial functional genes was calculated as the ratio of the copy number of each microbial functional gene to the samples corresponding 16S rRNA gene copy number.

Table S1. Primer sequences for PCR

Antibiotic	Gene	Amplicon Length (bp)	Forward Primers (5'-3')	Reverse Primers (5'-3')
16S rRNA gene	*16S*	60	GGGTTGCGCTCGTTGC	ATGGYTGTCGTCAGCTCGTG
Tetracycline	*tetK*	145	CAGCAGTCATTGGAAAATTATCTGATTATA	CCTTGTACTAACCTACCAAAAATCAAAATA
	tetQ	98	CGCCTCAGAAGTAAGTTCATACACTAAG	TCGTTCATGCGGATATTATCAGAAT
	tetW	100	ATGAACATTCCCACCGTTATCTTT	ATATCGGCGGAGAGCTTATCC
	tetO	104	CAACATTAACGGAAAGTTTATTGTATACCA	TTGACGCTCCAAATTCATTGTATC
	tetX	91	AAATTTGTTACCGACACGGAAGTT	CATAGCTGAAAAAATCCAGGACAGTT
	tetC	105	ACTGGTAAGGTAAACGCCATTGTC	ATGCATAAACCAGCCATTGAGTAAG
	tetE	64	TTGGCGCTGTATGCAATGAT	CGACGACCTATGCGATCTGA
	tetD	215	AATTGCACTGCCTGCATTGC	GACAGATTGCCAGCAGCAGA
	tetM	158	GGAGCGATTACAGAATTAGGAAGC	TCCATATGTCCTGGCGTGTC
Sulfonamide	*strB*	91	GCTCGGTCGTGAGAACAATCT	CAATTTCGGTCGCCTGGTAGT
	sul1	102	GCCGATGAGATCAGACGTATTG	CGCATAGCGCTGGGTTTC

	sul2	95	TCATCTGCCAAACTCGTCGTTA	GTCAAAGAACGCCGCAATGT
Aminoglycoside	*aac(6')-Ib*	56	CGTCGCCGAGCAACTTG	CGGTACCTTGCCTCTCAAACC
	aacA/aphD	138	AGAGCCTTGGGAAGATGAAGTTT	TTGATCCATACCATAGACTATCTCATCA
	aadD	91	CCGACAACATTTCTACCATCCTT	ACCGAAGCGCTCGTCGTATA
	aadE	92	TACCTTATTGCCCTTGGAAGAGTTA	GGAACTATGTCCCTTTTAATTCTACAATCT
	strA	101	CCGGTGGCATTTGAGAAAAA	GTGGCTCAACCTGCGAAAAG
MLSB	*ermB*	135	GAACACTAGGGTTGTTCTTGCA	CTGGAACATCTGTGGTATGGC
	ermF	69	CAGCTTTGGTTGAACATTTACGAA	AAATTCCTAAAATCACAACCGACAA
	ermX	79	GCTCAGTGGTCCCCATGGT	ATCCCCCCGTCAACGTTT
	mefA	255	TAATTATCGCAGCAGCTGGTTC	GTTCCCAAACGGAGTATAAGAGTG
	mphA	206	TCAGCGGGATGATCGACTG	GAGGGCGTAGAGGGCGTA
Trimethoprim	*dfrA1*	85	GGAATGGCCCTGATATTCCA	AGTCTTGCGTCCAACCAACAG
	dfrA12	75	CCTCTACCGAACCGTCACACA	GCGACAGCGTTGAAACAACTAC
Vancomycin	*vanB*	68	TTGTCGGCGAAGTGGATCA	AGCCTTTTTCCGGCTCGTT
	vanC	67	CCTGCCACAATCGATCGTT	CGGCTTCATTCGGCTTGATA
	blaCT	91	GCGATAACGTGGCGATG	GTCGAGACGGAACGTTT

Beta Lactam	*X-M*		AAT	CGT
	blaTEM	239	CGCCGCATACACTATTCTCAG	GCTTCATTCAGCTCCGGTTC
Multidrug	*acrB*	64	AGTCGGTGTTCGCCGTTAAC	CAAGGAAACGAACGCAATACC
	oprD	50	ATGAAGTGGAGCGCCATTG	GGCCACGGCGAACTGA
	floR	129	AACCCGCCCTCTGGATCA	GCCGTCGAGAAGAAGACGAA
Fluoroquinolone	*qnrA*	114	AGGATTTCTCACGCCAGGATT	CCGCTTTCAATGAAACTGCAA
	qnrD	181	CGCTGGAATGGCACTGTGA	GCTCTCCATCCAACTTCACTCC
	qnrS2	148	TCCCGAGCAAACTTTGCCAA	GGTGAGTCCCTATCCAGCGA
Others	*mcr-1*	269	CACATCGACGGCGTATTCTG	CAACGAGCATACCGACATCG
	optrA	155	GGTGGATGAAGTCCGTACGG	AGGTTAGACCTCCAAGAGCCA
MGEs	*intI1*	196	GCCTTGATGTTACCCGAGAG	GATCGGTCGAATGCGTGT
	intI2	195	TGCTTTTCCCACCCTTACC	GACGGCTACCCTCTGTTATCTC
	ISCR1	475	ATGGTTTCATGCGGGTT	CTGAGGGTGTGAGCGAG
	IS26	275	ATGGATGAAACCTACGTGAAGGTC	CGGTACTTAATCTGTCGGTGTTCA
	IS613	101	AGGTTCGGACTCAATGCAACA	TTCAGCACATACCGCCTTGAT

Table S2. The absolute quantification of ARGs, MGEs and 16S rRNA genes

	RM		CK						LCP						HCP					
gene	RM	SD (n=3)	3d	SD (n=3)	10d	SD (n=3)	30d	SD (n=3)	3d	SD (n=3)	10d	SD (n=3)	30d	SD (n=3)	3d	SD (n=3)	10d	SD (n=3)	30d	SD (n=3)
aac(6')-Ib	1.12E+09	1.45E+08	1.46E+07	1.85E+06	5.54E+06	1.51E+06	1.98E+06	1.45E+06	4.03E+07	2.18E+07	1.08E+07	2.87E+06	9.12E+06	1.37E+06	8.66E+06	2.34E+06	1.36E+07	2.40E+06	1.80E+06	6.63E+05
aacA/aphD	9.24E+06	5.24E+06	1.93E+06	1.73E+05	3.15E+06	1.99E+06	0.00E+00	0.00E+00	8.34E+05	3.84E+05	4.44E+05	3.97E+05	6.77E+05	6.17E+05	8.58E+05	1.87E+05	3.05E+05	2.90E+05	6.91E+05	6.43E+05
aadD	3.31E+06	1.22E+06	1.45E+07	9.32E+05	2.80E+06	1.28E+05	7.70E+05	8.62E+05	1.94E+07	7.14E+06	3.91E+05	3.63E+05	8.85E+05	9.31E+05	1.31E+07	5.05E+06	6.18E+05	5.53E+05	1.46E+06	1.67E+05
aadE	5.08E+07	2.84E+06	3.05E+07	5.61E+06	1.00E+07	3.20E+06	2.87E+06	6.93E+05	2.28E+07	1.84E+06	1.07E+07	3.21E+06	1.26E+07	9.99E+06	5.42E+06	3.19E+05	7.22E+05	8.36E+04	1.27E+06	3.85E+05
acrB	4.11E+07	3.27E+06	4.91E+06	3.53E+05	4.02E+06	1.18E+06	4.69E+05	3.99E+05	2.62E+05	4.90E+04	1.69E+06	5.56E+05	5.01E+05	4.85E+05	3.35E+04	3.19E+04	1.18E+05	1.32E+05	2.45E+05	2.31E+05
blaCTX-M	8.68E+05	1.03E+06	3.62E+04	3.14E+04	3.02E+05	2.62E+05	2.06E+05	3.87E+04	0.00E+00	0.00E+00	2.40E+05	8.55E+04	5.71E+04	9.88E+04	0.00E+00	0.00E+00	6.65E+04	6.46E+04	1.02E+05	1.01E+05
blaTEM	8.90E+08	1.08E+08	6.43E+06	1.74E+06	2.03E+07	9.16E+06	4.36E+06	3.30E+06	2.94E+06	9.18E+05	1.67E+07	5.74E+06	7.14E+06	2.52E+06	6.24E+05	2.68E+05	8.30E+05	5.68E+04	2.74E+06	3.46E+05

dfrA1	4.52E+08	5.74E+07	4.86E+06	1.30E+06	1.57E+06	4.31E+05	3.37E+05	2.93E+05	8.61E+05	3.52E+05	3.72E+06	6.81E+05	1.37E+07	1.24E+07	2.48E+06	1.19E+06	1.68E+06	6.10E+05	1.17E+06	3.51E+05
dfrA12	1.36E+08	2.00E+07	7.68E+06	3.82E+05	2.63E+07	8.58E+06	1.28E+07	3.55E+06	7.62E+05	1.65E+05	4.16E+06	1.68E+06	1.41E+07	1.36E+07	4.95E+06	2.52E+06	1.16E+06	7.32E+04	2.87E+06	4.79E+05
ermB	1.07E+08	1.72E+07	2.05E+06	1.56E+05	6.99E+05	3.83E+05	0.00E+00	0.00E+00	1.50E+06	9.07E+05	8.99E+05	2.12E+05	8.94E+05	8.47E+05	3.46E+06	1.03E+06	1.47E+06	7.95E+05	3.31E+06	4.48E+05
ermF	3.39E+09	9.35E+07	3.91E+06	2.85E+05	2.25E+07	3.77E+05	5.65E+06	1.96E+06	6.50E+06	2.65E+06	1.31E+08	3.23E+07	1.07E+08	2.25E+07	2.03E+06	6.37E+05	7.64E+06	5.55E+05	3.58E+06	1.22E+06
ermX	3.61E+07	3.36E+06	4.48E+06	2.46E+05	9.56E+05	1.08E+05	0.00E+00	0.00E+00	3.51E+06	1.82E+06	5.11E+06	1.49E+06	3.91E+06	2.42E+06	1.64E+06	7.02E+05	8.06E+05	7.80E+05	1.81E+06	3.21E+05
floR	1.03E+10	8.32E+08	4.41E+07	1.40E+06	3.07E+08	3.20E+07	2.63E+08	1.76E+08	1.28E+07	5.03E+06	4.36E+07	9.16E+06	2.14E+07	6.16E+06	5.36E+06	1.77E+06	1.69E+07	5.71E+06	8.26E+06	7.18E+05
mcr-1	1.75E+05	1.52E+05	0.00E+00	0.00E+00	7.93E+04	6.87E+04	0.00E+00	0.00E+00	0.00E+00	0.00E+00	0.00E+00	0.00E+00	0.00E+00	0.00E+00	0.00E+00	0.00E+00	6.24E+04	5.41E+04	1.06E+05	9.20E+04
mefA	6.27E+06	1.19E+06	3.46E+05	2.28E+05	0.00E+00	0.00E+00	0.00E+00	0.00E+00	7.49E+05	2.57E+05	0.00E+00	0.00E+00	1.62E+05	1.40E+05	3.24E+05	3.33E+05	5.71E+05	5.52E+05	1.95E+05	1.71E+05
mphA	1.87E+07	7.86E+04	1.53E+05	2.49E+04	1.00E+06	6.75E+05	4.21E+05	1.17E+05	2.74E+05	2.93E+05	3.35E+05	2.96E+05	6.16E+05	5.74E+05	6.08E+04	5.27E+04	1.09E+05	9.84E+04	5.28E+05	4.74E+05

oprD	4.03E+07	2.06E+05	1.13E+06	4.78E+05	5.92E+06	1.73E+06	9.96E+06	9.22E+06	3.04E+05	1.88E+05	8.46E+06	1.91E+06	1.79E+06	9.51E+05	1.54E+05	1.87E+05	2.15E+06	6.69E+05	1.27E+06	5.38E+05
optrA	1.51E+07	4.12E+06	2.16E+05	5.75E+04	0.00E+00	0.00E+00	1.31E+05	1.34E+05	1.40E+05	1.61E+05	3.92E+05	3.65E+05	0.00E+00	0.00E+00	3.74E+05	1.18E+05	8.69E+04	1.51E+05	7.52E+04	6.65E+04
qnrA	0.00E+00	0.00E+00	0.00E+00	0.00E+00	0.00E+00	0.00E+00	0.00E+00	0.00E+00	5.92E+04	5.20E+04	0.00E+00	0.00E+00	0.00E+00	0.00E+00	0.00E+00	0.00E+00	0.00E+00	0.00E+00	0.00E+00	0.00E+00
qnrD	7.43E+07	4.39E+06	6.11E+06	5.13E+05	1.56E+07	4.30E+06	8.86E+06	4.88E+06	6.18E+06	5.38E+05	2.99E+07	7.96E+06	1.26E+07	4.08E+06	3.02E+06	8.79E+05	7.67E+06	1.95E+06	1.95E+07	3.53E+06
qnrS2	1.71E+06	8.23E+04	2.43E+05	9.19E+04	0.00E+00	0.00E+00	0.00E+00	0.00E+00	0.00E+00	0.00E+00	1.15E+05	1.00E+05	0.00E+00	0.00E+00	0.00E+00	0.00E+00	0.00E+00	0.00E+00	0.00E+00	0.00E+00
strA	9.90E+07	7.95E+06	3.63E+06	4.51E+05	2.96E+07	1.04E+07	8.85E+05	5.16E+05	2.92E+06	2.11E+06	1.60E+06	4.21E+05	2.24E+06	1.14E+06	1.22E+06	2.77E+05	1.44E+06	8.92E+05	1.17E+06	6.83E+05
strB	4.78E+09	6.89E+08	1.23E+08	7.71E+06	2.68E+08	9.07E+06	1.06E+08	5.53E+07	6.33E+07	1.56E+07	5.87E+07	1.38E+07	3.41E+07	8.22E+06	9.51E+06	2.80E+06	3.18E+07	1.92E+06	2.00E+07	3.20E+06
sul1	5.49E+09	5.90E+08	5.49E+08	5.28E+07	7.81E+08	3.06E+07	2.99E+08	1.42E+08	3.47E+08	1.11E+08	8.27E+08	2.39E+08	4.71E+08	8.24E+07	1.84E+08	2.70E+07	5.24E+08	2.62E+07	1.65E+08	1.27E+07
sul2	7.35E+09	6.12E+08	3.24E+07	1.65E+06	1.70E+08	1.31E+07	1.26E+08	5.92E+07	1.27E+07	6.21E+06	6.52E+07	1.28E+07	6.89E+07	1.27E+07	3.41E+06	6.12E+05	3.50E+07	1.74E+06	4.06E+07	8.21E+06

tetC	2.43E+07	1.33E+06	1.93E+06	6.25E+05	2.84E+06	1.90E+06	3.36E+04	3.20E+04	3.20E+05	2.80E+05	1.74E+06	8.62E+05	2.98E+05	5.15E+05	2.77E+04	2.44E+04	1.36E+05	1.51E+05	0.00E+00	0.00E+00
tetD	3.31E+07	9.20E+06	2.64E+05	3.25E+04	1.69E+06	7.58E+05	3.61E+06	2.47E+06	7.07E+05	4.51E+05	2.35E+06	9.91E+05	1.44E+07	6.98E+06	2.13E+05	1.85E+05	3.91E+05	4.10E+05	2.16E+06	1.23E+06
tetE	3.58E+05	3.11E+05	6.57E+04	5.73E+04	0.00E+00	0.00E+00	1.04E+05	9.16E+04	1.63E+05	1.52E+05	2.23E+05	1.95E+05	0.00E+00	0.00E+00	1.99E+04	3.45E+04	7.88E+04	2.75E+03	0.00E+00	0.00E+00
tetK	1.22E+05	1.06E+05	4.16E+05	1.85E+05	6.02E+06	4.34E+06	1.16E+04	2.02E+04	3.45E+05	8.28E+04	0.00E+00	0.00E+00	1.68E+05	1.45E+05	1.80E+05	1.61E+05	1.13E+06	7.06E+05	4.87E+04	8.44E+04
tetM	7.37E+08	5.95E+07	4.90E+06	1.51E+06	2.64E+06	7.16E+05	1.18E+06	9.05E+05	1.15E+07	7.99E+06	1.06E+06	4.87E+05	7.84E+04	1.36E+05	4.23E+06	3.64E+06	1.59E+06	7.06E+04	1.64E+05	4.38E+04
tetO	7.00E+06	3.27E+06	5.57E+05	8.39E+04	0.00E+00	0.00E+00	2.51E+05	1.86E+05	4.84E+05	4.25E+05	3.65E+05	6.32E+05	3.28E+05	5.68E+05	8.08E+05	3.96E+05	1.02E+05	8.83E+04	5.42E+05	4.70E+05
tetQ	1.84E+08	3.00E+06	4.77E+07	1.17E+07	1.22E+08	4.15E+07	6.74E+07	3.69E+07	2.91E+07	2.29E+06	2.04E+08	4.99E+07	1.19E+08	5.27E+07	4.54E+06	4.66E+06	6.77E+07	2.54E+07	1.39E+08	3.80E+07
tetW	3.75E+07	5.23E+05	1.04E+06	2.39E+05	2.19E+06	1.90E+06	4.00E+05	4.11E+05	1.55E+06	1.16E+06	0.00E+00	0.00E+00	6.49E+05	5.70E+05	8.47E+05	8.70E+05	4.57E+05	3.97E+05	6.39E+05	6.22E+05
tetX	9.18E+09	5.18E+08	6.23E+07	1.94E+07	7.77E+08	1.70E+08	3.66E+08	1.27E+08	4.77E+07	1.21E+07	6.08E+08	2.04E+08	3.93E+08	1.37E+08	8.18E+06	2.16E+06	2.00E+08	5.58E+07	1.49E+08	3.87E+07

vanB	4.68E+05	4.07E+05	2.72E+04	2.37E+04	5.59E+04	4.85E+04	0.00E+00	0.00E+00	7.95E+04	1.88E+04	0.00E+00	0.00E+00	2.56E+05	2.28E+05	3.20E+04	2.80E+04	0.00E+00	0.00E+00	3.04E+05	4.28E+05
vanC	2.42E+05	3.08E+04	0.00E+00	0.00E+00	2.31E+05	2.00E+05	0.00E+00	0.00E+00	9.50E+04	8.71E+04	2.07E+05	1.85E+05	5.76E+04	5.26E+04	4.35E+04	4.75E+04	8.60E+04	3.41E+04	2.08E+05	1.81E+05
intI1	3.05E+09	1.81E+08	4.13E+07	5.39E+06	1.04E+08	2.44E+07	3.97E+07	2.37E+07	3.27E+07	7.87E+06	1.96E+07	5.40E+06	2.81E+07	4.14E+06	8.43E+06	4.63E+05	1.03E+07	1.27E+06	9.93E+06	2.10E+06
intI2	4.16E+08	7.80E+07	2.71E+05	2.34E+05	1.04E+06	9.03E+05	3.43E+05	3.63E+05	2.19E+06	1.12E+06	1.96E+06	1.08E+06	1.88E+05	1.77E+05	1.91E+05	1.67E+05	4.96E+05	4.82E+05	1.75E+05	1.52E+05
IS26	8.40E+09	1.88E+09	4.95E+07	2.53E+06	1.65E+08	3.22E+07	4.94E+07	2.89E+07	1.14E+07	7.26E+06	2.10E+08	7.59E+07	3.31E+07	1.11E+07	8.90E+06	2.20E+06	6.66E+07	6.85E+06	1.33E+07	2.57E+06
IS613	2.37E+07	4.74E+06	7.85E+04	4.87E+04	0.00E+00	0.00E+00	1.06E+05	1.01E+05	2.34E+05	2.06E+05	2.30E+05	2.16E+05	4.27E+05	5.13E+05	2.21E+04	1.93E+04	0.00E+00	0.00E+00	3.23E+05	3.00E+05
ISCR1	4.36E+07	2.49E+06	0.00E+00	0.00E+00	7.20E+05	6.26E+05	4.13E+05	3.69E+05	0.00E+00	0.00E+00	0.00E+00	0.00E+00	0.00E+00	0.00E+00	1.52E+04	1.36E+04	3.74E+05	3.25E+05	5.24E+05	4.63E+05
16SrRNA	3.86E+10	2.11E+09	9.25E+09	1.11E+09	1.57E+10	6.13E+08	8.97E+09	4.51E+09	6.80E+09	2.00E+09	1.61E+10	4.97E+09	1.82E+10	4.02E+09	3.52E+09	4.70E+08	1.01E+10	7.79E+08	1.27E+10	2.09E+09

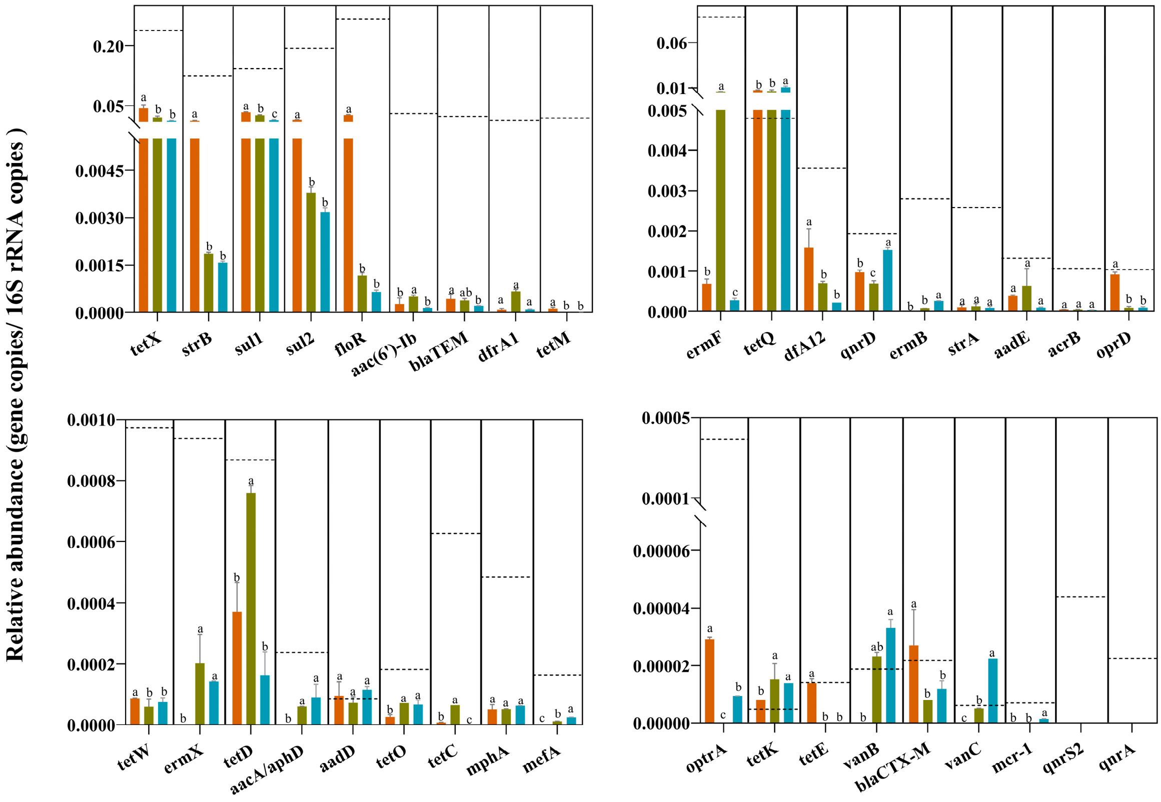

Fig. S1. RAs of each ARGs after composting. The dotted line represents the RAs of each gene at the start of co-composting. Bars denote standard errors.

a-c Within an ARG, treatments without a common superscript differed (*P*<0.05).

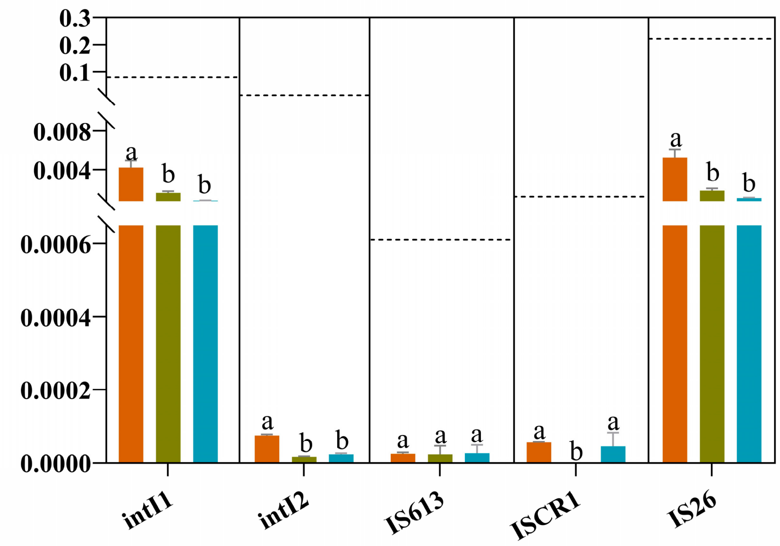

Fig. S2. RAs of each MGEs after composting. The dotted line represents the RAs of each gene at the start of co-composting. Bars denote standard errors.

a-c Within an ARG, treatments without a common superscript differed (*P*<0.05).

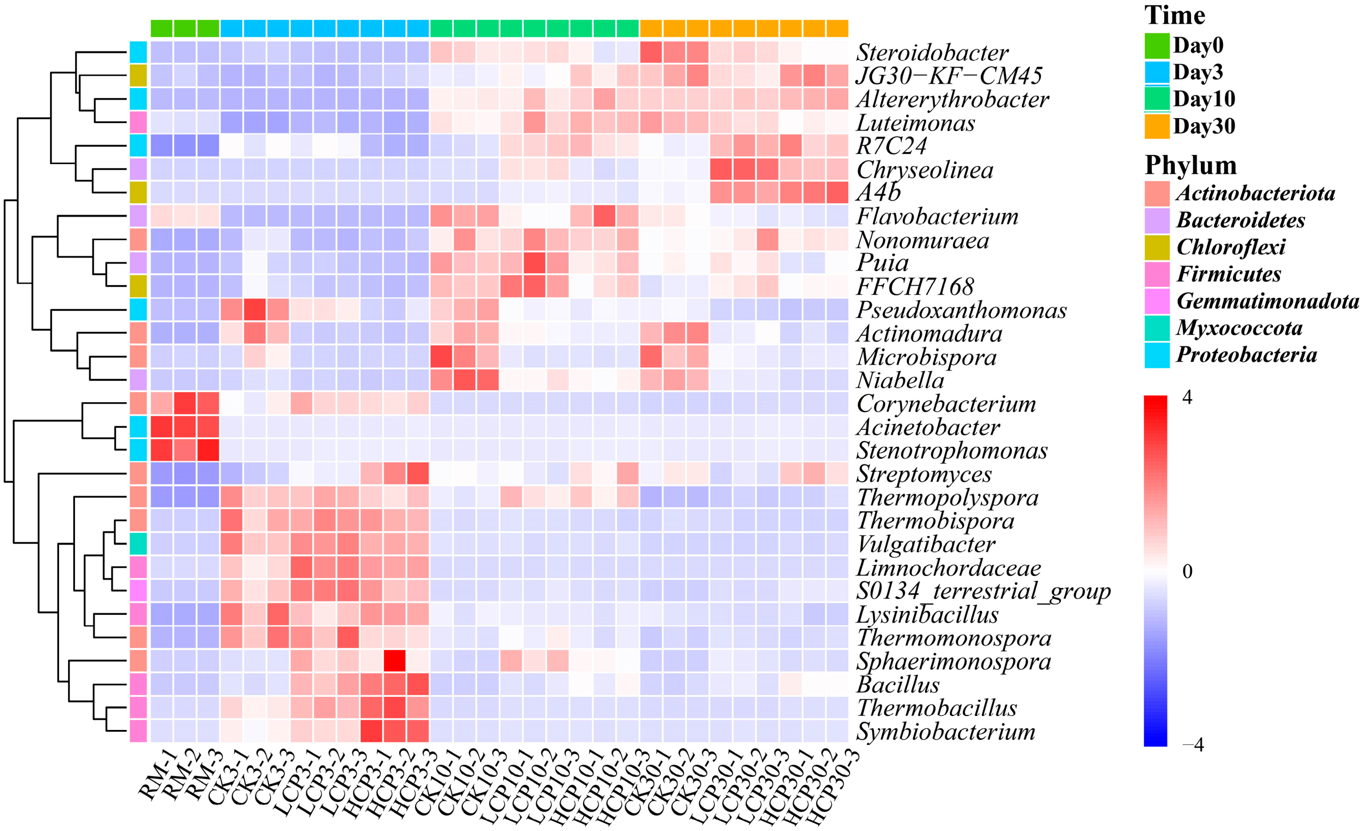

Fig. S3. Heatmap showing relative abundances of the top 30 genera in all samples.

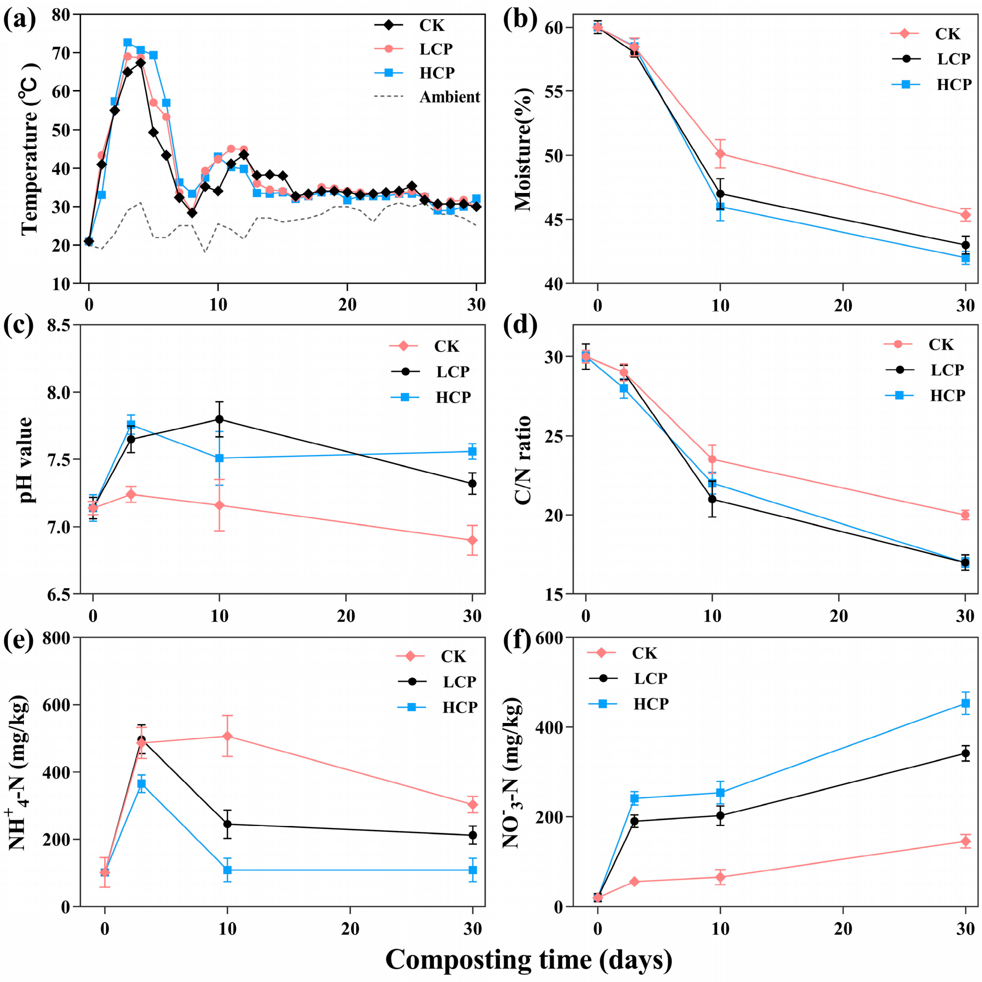

Fig. S4. Changes in the physicochemical properties during composting. (a) temperature, (b) moisture, (c) pH value, (d) C/N ratio, (e) $NH^{+}{}_{4}$-N, (f) $NO^{-}{}_{3}$-N

References

[1] B. Zheng, Y. Zhu, J. Sardans, J. Peñuelas, J. Su, QMEC: a tool for high-throughput quantitative assessment of microbial functional potential in C, N, P, and S biogeochemical cycling. Sci. China Life Sci. 61 (2018), 1451-1462. https://doi.org/10.1007/s11427-018-9364-7.

[2] Y. Ouyang, G. R. Duarte, B. L. Poe, P. S. Riehl, F. M. dos Santos, C. C. Martin-Didonet, E. Carrilho, J. P. Landers, A disposable laser print-cut-laminate polyester microchip for multiplexed PCR via infra-red-mediated thermal control, Anal. Chim. Acta. 901 (2015), 59-67. https://doi.org/10.1016/j.aca.2015.09.042.
